# Correction of Linezolid-Induced Myelotoxicity After Switch to Tedizolid in a Patient Requiring Suppressive Antimicrobial Therapy for Multidrug-Resistant *Staphylococcus epidermidis* Prosthetic-Joint Infection

**DOI:** 10.1093/ofid/ofy246

**Published:** 2018-09-25

**Authors:** Tristan Ferry, Cécile Batailler, Anne Conrad, Claire Triffault-Fillit, Frédéric Laurent, Florent Valour, Christian Chidiac, Lyon BJI Study Group, Tristan Ferry, Florent Valour, Thomas Perpoint, André Boibieux, François Biron, Patrick Miailhes, Florence Ader, Agathe Becker, Sandrine Roux, Claire Triffault-Fillit, Anne Conrad, Alexie Bosch, Fatiha Daoud, Johanna Lippman, Evelyne Braun, Christian Chidiac, Sébastien Lustig, Elvire Servien, Romain Gaillard, Antoine Schneider, Stanislas Gunst, Cécile Batailler, Michel-Henry Fessy, Yannick Herry, Anthony Viste, Philippe Chaudier, Cyril Courtin, Lucie Louboutin, Sébastien Martres, Franck Trouillet, Cédric Barrey, Francesco Signorelli, Emmanuel Jouanneau, Timothée Jacquesson, Ali Mojallal, Fabien Boucher, Hristo Shipkov, Joseph Chateau, Frédéric Aubrun, Mikhail Dziadzko, Caroline Macabéo, Frederic Laurent, Céline Dupieux, Jérôme Josse, Camille Kolenda, Fabien Craighero, Loic Boussel, Jean-Baptiste Pialat, Isabelle Morelec, Marc Janier, Francesco Giammarile, Michel Tod, Marie-Claude Gagnieu, Sylvain Goutelle, Eugénie Mabrut

**Affiliations:** 1 Service de Maladies Infectieuses, Hôpital de la Croix-Rousse, Hospices Civils de Lyon, France; 2 Université Claude Bernard Lyon 1, France; 3 Centre International de Recherche en Infectiologie, CIRI, Inserm U1111, CNRS UMR5308, ENS de Lyon, UCBL1, France; 4 Centre Interrégional de Référence des Infections Ostéo-articulaires Complexes (CRIOAc Lyon), Hospices Civils de Lyon, France; 5 Service de Chirurgie Orthopédique, Hôpital de la Croix-Rousse, Hospices Civils de Lyon, France; 6 Laboratoire de Bactériologie, Institut des Agents Infectieux, Hôpital de la Croix-Rousse, Hospices Civils de Lyon, France

A 71-year-old man (85 kg) has a past history of vitiligo, ischemic myocardiopathy, and bilateral knee arthroplasties. A 1-stage exchange of the right prosthetic-joint infection (PJI) was done in 2016 for a mechanical prosthetic loosening. A massive constrained prosthetic joint was used to compensate for the bone loss (Supplementary Figure S1A). Iterative postoperative dislocations were followed by occurrence of a fistula in January 2017 and prosthetic loosening (Supplementary Figure S1B) without any pain. Because it was impossible to imagine a 2-stage exchange, a debridement and implant retention (DAIR) procedure followed by suppressive antimicrobial therapy was proposed. Daptomycin (700 mg/day) and ceftaroline (1200 mg/day) were prescribed after the surgery. A multidrug-resistant *Staphylococcus epidermidis*, which is only susceptible to daptomycin, vancomycin, fosfomycin, and linezolid, was found in culture from all operative samples. After 6 weeks of intravenous antimicrobial therapy, 600 mg of linezolid bid was prescribed in August 2017. Concomitant medications were ramipril, bisoprolol, furosemide, and aspirin. Under therapy, the patient experienced a progressive decrease of hemoglobin and hematocrit (without decrease of white blood cells or platelets). Five months after linezolid introduction, the patient developed asthenia related to anemia, with a decrease of hemoglobin to 65 mg/dL, and without leucopenia or thrombocytopenia (Figure 1). The patient did not take any treatment with potential bone marrow toxicity, except linezolid. The patient has no other adverse drug reactions. A blood transfusion (2 bags) was performed, which led to an immediate increase of the hemoglobin level to 84 mg/dL, and linezolid was switched to 200 mg of tedizolid once a day. In May 2018, 9 months after the DAIR surgery and 4 months after the switch, the patient was perfectly fine, walked despite rupture of the right knee extensor apparatus (video S2), without any pain, without any local signs of relapse (Supplementary Figure S1C), without clinical signs of neuropathy, and he experienced a continuous increase of the hemoglobin to 14 mg/dL under tedizolid therapy. No other treatment was changed or introduced.

**Figure 1.**
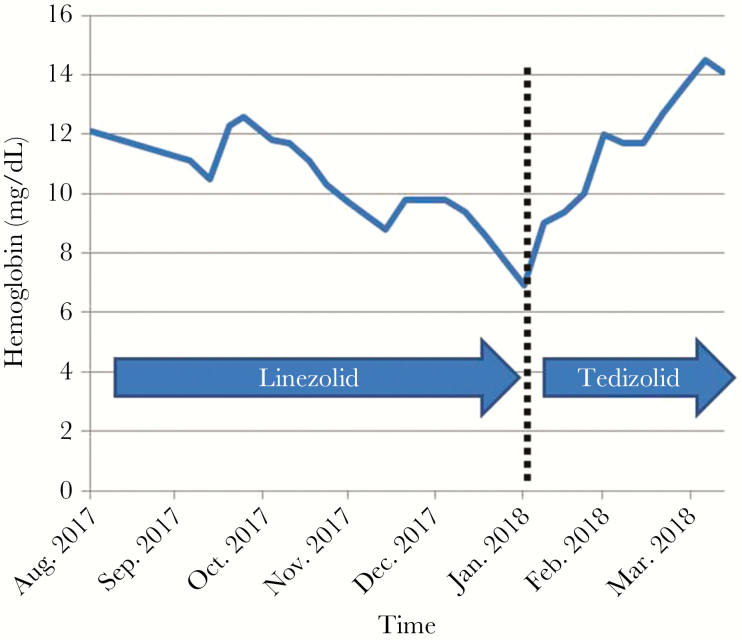
Hemoglobin during time, with continuous decrease under linezolid therapy, followed by a continuous increase after the switch to tedizolid.

Suppressive antimicrobial therapy (ie, indefinite chronic oral treatment with antibiotics) is an option for patients with chronic PJI, especially for elderly patients [1, 2]. The decision to offer chronic suppressive therapy must take into account the individual circumstances of the patient including the functional status, the risk of a further exchange revision, the risk of amputation, and the drug susceptibility of the pathogen. The most frequently used drugs are amoxicillin (mainly for streptococcal and enterococcal PJI), sulphamethoxazole-trimethoprim, fluoroquinolones, and doxycycline (mainly for staphylococcal and *Enterobacteriaceae* PJI) [1–3]. In patients with multidrug-resistant staphylococcal PJI, there are often very few options, including off-labeled antibiotics such as pristinamycin or linezolid. Pristinamycin in an oral streptogramin antibiotic made up of 2 synergistic but structurally unrelated components, pristinamycin IA and pristinamycin IIA, that remains active on multidrug-resistant staphylococci [2, 4]. Pristinamycin, available only in France and in Australia, needs at least 3 doses in a day, and it is frequently associated with a poor digestive tolerance. We can also imagine that intravenous dalbavancin, with injection every 8 weeks as suppressive therapy, could be an emergent treatment option in such patients in the future. Linezolid is an oxazolidinone drug active against a wide range of Gram-positive bacteria, but it induced myelotoxicity including anemia and thrombocytopenia after weeks to months of treatment. Tedizolid is a new oxazolidinone drug currently approved for the treatment of skin and soft tissue infections, at the dose of 200 mg once a day for 6 days. Based on the results of the clinical trials that assessed the noninferiority of tedizolid (200 mg/day for 6 days) versus linezolid (600 mg bid during 10 days) in acute skin and soft tissue infections, a lower incidence of myelotoxicity was observed [5]. Moreover, preclinical data showed a potential for a lower toxicity, which could be explained by the once-daily administration, that might allow a recovery period in mitochondrial toxicity [6, 7]. Few case reports (n = 5) have been published about patients who received prolonged tedizolid treatment. Those patients were treated for mycobacterial infections, abscesses, or chronic vascular graft infections [8–10]. To the best of our knowledge, this is the first case of a patient with PJI receiving prolonged tedizolid as suppressive therapy. Our patient presented with recurrent multidrug-resistant *S epidermidis* PJI, and oxazolidinone was the only treatment option. Because the patient developed significant myelotoxicity, we switched from linezolid to tedizolid and the myelotoxicity reversed, without any clinical signs of toxicity. This phenomenon has already been described in patients receiving tedizolid after linezolid, although a subsequent decline of hemoglobin level leading to tedizolid interruption was also observed in 1 patient [8].

## CONCLUSIONS

Because tedizolid is an easy-to-take drug that may have less myelotoxicity potential than linezolid, it is a promising option for patients with multidrug-resistant staphylococci who require suppressive antimicrobial therapy. However, the limited experience with this drug and the very high price of the molecule (1 year’s supply of tedizolid is approximately $127000 in the United States, €75000 in France) limit the large use of tedizolid in this indication.

## Supplementary Data

Supplementary materials are available at *Open Forum Infectious Diseases* online. Consisting of data provided by the authors to benefit the reader, the posted materials are not copyedited and are the sole responsibility of the authors, so questions or comments should be addressed to the corresponding author.

ofy246_suppl_supplementary_video

ofy246_suppl_supplementary_diapo_1
