# Supplementary figures and images for: Correction of Linezolid-Induced Myelotoxicity After Switch to Tedizolid in a Patient Requiring Suppressive Antimicrobial Therapy for Multidrug-Resistant Staphylococcus epidermidis Prosthetic-Joint Infection
Source: Open Forum Infect Dis. 2018 Sep 25;5(10):ofy246. doi: 10.1093/ofid/ofy246 (PMC6198638; doi:10.1093/ofid/ofy246)

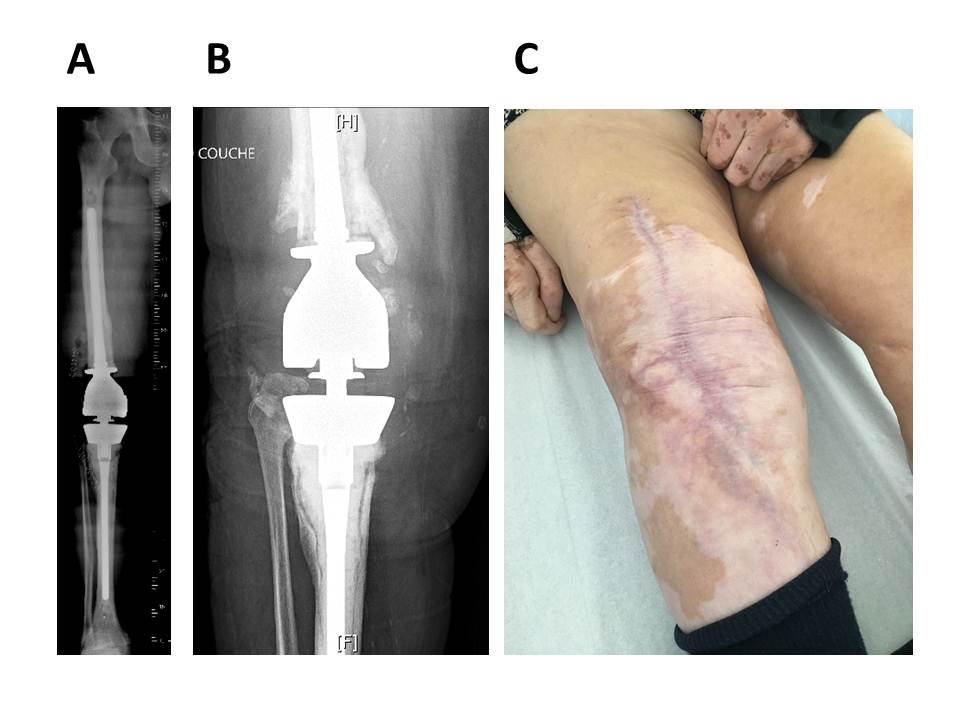

Supplement: ofy246_suppl_supplementary_diapo_1 [file ofy246_suppl_supplementary_diapo_1.jpeg]
